# The role of primary care in adult weight management: qualitative interviews with key stakeholders in weight management services

**DOI:** 10.1186/s12913-017-2729-7

**Published:** 2017-11-21

**Authors:** David N. Blane, Sara Macdonald, David Morrison, Catherine A. O’Donnell

**Affiliations:** 1 General Practice and Primary Care, Institute of Health and Wellbeing, University of Glasgow, 1 Horselethill Road, Glasgow, G12 9LX UK; 2 Public Health, Institute of Health and Wellbeing, University of Glasgow, 1 Lilybank Gardens, Glasgow, G12 8RZ UK

**Keywords:** Obesity, Primary care, Health services, Weight management, Qualitative

## Abstract

**Background:**

Primary care has a key role to play in the prevention and management of obesity, but there remain barriers to engagement in weight management by primary care practitioners. The aim of this study was to explore the views of key stakeholders in adult weight management services on the role of primary care in adult weight management.

**Methods:**

Qualitative study involving semi-structured interviews with nine senior dietitians involved in NHS weight management from seven Scottish health boards. Transcripts were analysed using an inductive thematic approach.

**Results:**

A range of tensions were apparent within three key themes: weight management service issues, the role of primary care, and communication with primary care. For weight management services, these tensions were around funding, the management model of obesity, and how to configure access to services. For primary care, they were around *what* primary care should be doing, *who* should be doing it, and *where* this activity should fit within wider weight management policy. With regard to communication between weight management services and primary care, there were tensions related to the *approach* taken (locally adapted versus centralised), the *message* being communicated (weight loss versus wellbeing), and the *response* from practitioners (engagement versus resistance).

**Conclusions:**

Primary care can do more to support adult weight management, but this requires better engagement and communication with weight management services, to overcome the tensions highlighted in this study. This, in turn, requires more secure, sustained funding. The example of smoking cessation in the UK, where there is a network of well-resourced NHS Stop Smoking Services, accessible via different means, could be a model to follow.

**Electronic supplementary material:**

The online version of this article (10.1186/s12913-017-2729-7) contains supplementary material, which is available to authorized users.

## Background

Current United Kingdom (UK) guidelines on obesity emphasise a key role for primary care, particularly in the identification of individuals with obesity and appropriate signposting or referral to weight management services. [1, 2]. The strengths of primary care – population coverage, contact, and continuity [3] – support this role in theory, but there is a considerable gap between policy rhetoric (“*every healthcare contact is a health improvement opportunity*” [4]) and the reality in practice. Obesity remains under-treated in primary care: few patients are referred to external sources of support, where they exist, and there are wide variations in referral rates and attendance following referral [5, 6].

Previous research has explored the barriers to engagement with weight management from the perspective of primary care practitioners (i.e. general practitioners (GPs) and practice nurses). This identified: lack of time in the consultation [7]; lack of knowledge and lack of confidence in discussing weight [8]; perceptions of poor outcomes of interventions [8]; fear of causing offence [9]; and a belief that individuals are responsible for obesity and it’s not a medical problem [10]. There has, however, been a paucity of research exploring the views of those senior professionals – usually dietitians by background – involved in the strategic planning and delivery of adult weight management services [11]. In particular, understanding their views on the role of primary care and how they have engaged with primary care practitioners may help us to improve communication and referrals between services, and ultimately improve adult weight management.

The recent BWeL study showed that a brief intervention by GPs, offering referral to a local weight management service, was both acceptable and effective [12]. The authors argued that if National Health Service (NHS) weight management services were resourced to the same extent as smoking cessation services, then this would increase the impact that primary care can have on population obesity levels [13]. The ‘change fatigue’ that referring practitioners experience when services are constantly changing would be less of an issue [14], and access to weight management services would improve.

The NHS in Scotland is publicly funded (largely through taxation) and there are 14 regional NHS Health Boards that are responsible for the delivery of all frontline healthcare services, including adult weight management. In theory, NHS weight management services in Scotland are based around a comprehensive tiered approach, with Tier 1 representing community-based interventions such as walking groups or cooking classes, Tier 2 lifestyle interventions delivered in the community, Tier 3 specialist multi-disciplinary services (e.g. including physiotherapy and psychology) and Tier 4 bariatric surgery [15].

In practice, however, provision of weight management services is patchy and highly variable. A recent national survey of weight management provision in the 11 NHS health boards of mainland Scotland identified wide variation in the provision and access to services; only four health boards offered services for those with a body mass index (BMI) 25-30 kg/m^2^ and six health boards did not have both Tier 2 and Tier 3 services [16]. Some of the smaller health boards, such as the Orkney and Shetland Islands, do not have their own standalone weight management services, instead referring patients to one of the larger health boards. There is also variation in referral pathways to Tier 2 and 3 services, with some accepting self-referrals and others requiring GP referral. Tier 2 and 3 services are held in different health board locations across Scotland, including hospitals and health centres. This suggests a fluidity to the range of services and models available nationally which then have to interact with primary care.

The aim of this paper was to explore the views of key stakeholders involved in the planning and delivery of adult weight management services on the role of primary care in adult weight management and their experience of engaging with GPs and practice nurses.

## Methods

A qualitative approach was used, with semi-structured interviews chosen as the best approach for exploring the views and experiences of a purposive sample of stakeholders. Ethics approval was obtained through the University of Glasgow MVLS ethics committee (Project No: 200130121). An approved consent form was signed by each participant at the start of each interview after providing the opportunity to ask questions or, in the case of telephone interviews, was emailed or posted to the research team prior to the interview.

### Recruitment

Recruitment was by email to the service leads for adult weight management in all 14 health boards in Scotland, explaining the nature and purpose of the research. These individuals were targeted as they were assumed to have the necessary knowledge about the strategic planning and delivery of these services. The stakeholders that responded were from 7 of the 8 largest health boards, representing approximately 80% of the Scottish population. No attempts were made to contact non-respondents.

### Data collection

Seven interviews were conducted with nine interviewees between May and September 2014. Most interviews were conducted face-to-face, but three were conducted over the telephone and two conducted with two participants in a small group interview. The face-to-face interviews were held at venues arranged by the interviewees themselves, usually their place of work. DB, an academic general practitioner with previous experience of and training in qualitative research, conducted all interviews as part of his PhD research. SM, an experienced qualitative researcher with a background in sociology, was also present for the first three interviews. SM and DB discussed initial reflections after each interview, informing small changes to the interview topic guide (see Additional file 1). The topic guide included questions about the interviewee’s views on the role of primary care in adult weight management and their experience of engagement with primary care. It was influenced by Pawson’s idea of the ‘realist interview’ [17], as the interviews also informed a separate realist synthesis study [18]. Interviews lasted between 49 and 82 min, average 63.

### Data analysis

Interviews were audio-recorded and transcribed verbatim. The transcripts were then thoroughly checked for inconsistencies against the recordings and anonymised. QSR International NVIVO 10 qualitative data analysis software [19] was used to aid data handling and analysis.

The analysis process involved three steps, as described by Ziebland and McPherson [20]. The first step was coding. Initially, two transcripts were read closely and coded independently by DB, SM and COD. Coding clinics with DB, SM and COD were then held to review the codes for each of these transcripts and to agree on a coding framework. Subsequent transcripts were coded by DB according to this framework, with a further coding clinic to check the consistency of this coding.

The second step involved summarising the codes using the ‘OSOP’ (‘one sheet of paper’) method [20]. All the data contained within each main code was gathered in a report, reviewed and all the themes identified summarised on the eponymous sheet(s) of paper. There was similarity and repetition of themes across the nine interviewees, but it is hard to say if data saturation had occurred. The third step aimed to draw out ‘higher level’ explanations or links between the issues in an inductive thematic analysis [21].. These steps were led by DB in discussion with SM and COD.

## Results

### Interviewee characteristics

The nine interviewees were all experienced dietitians with senior positions related to weight management within their respective health boards. Most were either service leads, or were involved in policy, strategy, and service development for Tier 2 and/or 3 services. Table 1 summarises stakeholder characteristics.

**Table 1:** Interviewee characteristics

Interviewee code	Health Board Region	Description of Health Board Region	Adult weight management tiers and referral pathways
M1^a^ F1	A	Large^b^, Urban	2 – GP referral 3 – GP referral
F2	B	Medium, Mixed Rural/Urban	2 – Self-referral 3 – No service
F3	C	Medium, Mixed Rural/Urban	2 – Mostly self-referral 3 – GP referral
F4	D	Medium, mostly Rural	2 – Mostly self-referral 3 – Pilot service (both)
F5	E	Large, Urban	2 – GP or secondary care referral 3 – GP or secondary care referral
M2 F6	F	Large, Urban	2 – Self-referral 3 – GP referral
F7	G	Medium, mostly Rural	2 – Dietetics or self-referral 3 – GP or secondary care referral

^a^ M = male; F = female

^b^Large is >600,000 population; Medium is 300-600,000 (mid-2014 estimates)

### Themes

There were three overarching themes that reflect the relationship between primary care and adult weight management services – issues related to weight management services themselves, the role of primary care in adult weight management, and communication with primary care. Within each of these, there were three sub-themes, framed as tensions that were evident in the data. The key themes are summarised in Table 2.

**Table 2:** Results of thematic analysis

Main theme	Sub-theme
Weight management service issues	*Mainstream* versus *Insecure funding* *Medical* versus *Social model* *Access* versus *Capacity*
Role of primary care	*Referral* versus *Signposting* *GP* versus *Practice nurse* *Practice* versus *Community level*
Communication with primary care	*Local* versus *Centralised models* *Weight loss* versus *Wellbeing messages* *Engagement* versus *Resistance*

### Weight management service issues

As noted, the interviewees worked in different health boards with differing approaches to adult weight management. One feature that was, however, consistent across most of the services was the struggle they had to secure funding. The tension between mainstream versus insecure funding is evident in the following quotes.

“We know for a fact that we will not have any physio input without funding, we won’t have any psychological input without funding and even simple things like venues and resources we are fairly limited for that as well.” (F2)

“My effort to get an NHS board to invest in adult weight management was, em, unsuccessful let’s say.” (F3)

A number of interviewees gave their views on why it was so hard to secure funding, which can be summed up as a lack of a coherent – and powerful – voice lobbying for resources.

“I find it all quite frustrating to be honest because I think it’s going back to… the fact it needs a very sort of cohesive group with somebody who has clout at the top and is able to get the argument for more resources to be put into weight management.” (F4)

The second tension related to weight management services was between applying a medical or social model to the management of obesity. On the one hand, interviewees recognised that the scale of overweight and obesity (affecting two-thirds of the adult population) is such that wider population measures need to be taken, but on the other hand the approaches used by the services were often individually-focussed, treating obesity as a chronic disease.

One health board, however, adopted a different model of weight management, following the principles of the *Health at every size (HAES)* movement [22]. This approach focusses on wellbeing rather than weight loss per se.

“In [health board G] we take a particular approach to weight management which isn’t about weight loss. In fact, we particularly, we try to get people to stop focussing on weight loss as a goal and look at health gain. So what is it about, the question we ask people, we say to people, ‘what is it about weight loss that’s important to you? And let’s work on that.’ So it might be that ‘I want to play with my grandchildren’, ‘I want to feel better about myself’, ‘I want to get my diabetes under control’, ‘I want to develop a better relationship with food’, you know. So that’s what we focus on.” (F7)

This represents a significant change of approach compared to other health boards in Scotland. It is the closest to a social model of obesity, with a focus on supporting patients *in their context* and challenging stigmatising societal attitudes to obesity*.*

The third tension was between a desire to make the service available to as many people as possible (i.e. widening access) and recognition that there was not enough capacity to support the potential numbers of eligible patients.

“When we set it up there was a lot of people around the table saying ‘we don’t want to promote this heavily because we think we are going to be inundated.’ We’ve not been...” (F2)

“We hadn’t actually gone out to GPs and said, ‘send us all your really overweight people’, because we were worried that would be overwhelming.” (F6)

Several approaches to the access versus capacity dilemma were described. The most common approach was the use of group sessions rather than one-to-one sessions for weight management classes.

“What has taken a lot of time to get engagement from our own, our own colleagues to do, is to apply a group approach because previous to that it was a one-to-one approach. They were able to show if nothing else from that is that on the basis of that one-to-one approach all they could address is 0.5% of need. A group approach we are now up to expecting to be able to address 2% of the need.” (F3)

Another approach was to work with local authorities or businesses to make use of their resources.

“In [health board F] we decided what we were going to do was we were going to upskill leisure colleagues, to deliver on our behalf.” (F6)

There were further considerations related to improving access to weight management services, which can be thought of in terms of both structure (e.g. location and timing) and process (e.g. self-referral or GP referral). The latter is explored in the next section.

### Role of primary care

Stakeholders expressed tensions about the role of primary care in adult weight management in three areas: *what* primary care should be doing, *who* should be doing it, and *where* this activity should fit in with wider weight management policy.

There was general agreement that primary care was not well placed to be *delivering* weight management interventions wholesale (i.e. structured courses of dietary advice, physical activity, psychological support, etc.), but that its focus should be on linking with weight management services. The first tension, therefore, was between signposting of patients to services versus formal referral. These appear very similar, both involving linking patients with a service, but for the interviewees they reflected differing attitudes to responsibility and risk. For those that advocated signposting, responsibility rests very much with the patient; once they have been told about a service and how to access it, it is up to them to make contact. Signposting, it is argued, encourages patient motivation more than the passive approach of *being referred*.

“I do think it should be, the onus should be on the person to think ‘right okay, that’s for me and I’m going to phone up about it and book myself onto a place’ rather than involving more paperwork, etc., etc., of a sort of formal referral going in.” (F4)

In contrast, those who advocated formal referral believed the GP ‘gatekeeper’ role was important, selecting those patients who may be most ‘appropriate’ for a weight management intervention.

“The model of care that we are providing in Tier two is, the gateway is the GP, so the GP will have identified with the patient and assessed their willingness, readiness to change.” (F1)

Furthermore, they highlighted the role of the GP in managing risk related to the referral, as this quote shows:

“So we got agreement from all the clinical leads that this question could be put on [electronic referral system] which runs through, the benefits of this – undertaking physical activity – outweigh the risks involved and there’s a big exclusion list and we got sign up that that is now on [electronic referral system], so that gives us assurance ‘well the GP has done that risk assessment’… so the GP is saying yes… so that gives us, well we can move ahead with our physical activity so I think that’s really important.” (M1)

Thus, some interviewees saw a clear role for GPs in risk assessment prior to referral. Others, though, felt that practice nurses were in a better position to engage with patients about weight management. The second tension, therefore, focussed on role remit and responsibility of GPs versus practice nurses.

“I think practice nurses think they have got more of a role in weight management in the talking to people and supporting people with their weight. I think in a traditional model a lot of the time might be that people come to see the practice nurse to get weighed because they know they have got a good set of scales.” (F7)

“I think it should be a routine part of care that there is a set of scales that you go on if you are coming to be treated for your blood pressure and you’re overweight, or your diabetes and you are overweight. Or your asthma and you are overweight, you know, it’s, practice nurses are in that routine and it's part of their care but I’m not sure if the GP would always do that.” (F1)

The third tension was between viewing primary care as a ‘hub’ of weight management activity or more of a peripheral player. It also relates to the extent to which general practices should be engaging with other community activities and services related to weight management, which ties in with the earlier tension between a medical or a social model of weight management.

“…part of this coming through that not to medicalise their weight problem too that there are other things that the patient should perhaps be given, steered into and, you know, I suppose that’s part of what our health and social care partnerships are about, trying to encourage more access to physical activity, healthier eating… and I think more and more general practitioners are trying to be, well part of the process and philosophy is to try and encourage those communities in the health centre so that there is more and more information available there that the patient can be, not directed, but you know, give them a steer towards and I think there is more of that going on now.” (F1)

The above quote reflects this tension and suggests that practices should be looking beyond their responsibilities to individual patients and be thinking more about their place within communities.

### Communication with primary care

Interviewees shared their experiences of working with primary care and how they have communicated with GPs and practice nurses. Again a series of tensions emerged. The first related to the *approach* taken to communication with primary care, between locally adapted versus more centralised models. The local models used more personal approaches to communication, such as face-to-face meetings with practitioners.

“We are starting to do, like, raising awareness sessions and just talking to some of the practice nurses in [health board B], you know they are quite interested in getting involved…” (F2)

In contrast, the more centralised models used more impersonal approaches such as various forms of electronic communication – email, website, intranet, or electronic newsletter. Of course, it is possible to use electronic communication in a personalised way – for instance, by providing practice-specific feedback on referrals by email – but this did not happen very often. Most services used a mixed model, with both central (impersonal) and local (personal) approaches.

“Each time the service moved out to a different [area] every practice was emailed and lettered with the referrals, information over here, and we also invited them to come here, or asked them if they’d like someone to come to the practice, and we’ve been to many practices.” (F5)

There was a feeling that in those areas where there was a previous history of working closely with practices (e.g. with a related service such as Exercise on Referral), the services benefitted from this improved relationship.

“What’s interesting is that where there has been long term sort of work between the local authorities and the GPs and practice nurses in the area they are getting much better referrals coming through. So where there is already a partnership, a relationship built up, they are getting, you know, they are getting frequent referrals coming through. In the areas where that’s not as well established then you can kind of see the difference.” (F2)

Method of communication was a key consideration. The more personal forms of communication were preferred by most, as the following quotes demonstrate.

“It’s very difficult sometimes to have a relationship with people if you have never actually met them, or the first time you are on the phone is to say ‘no I’m sorry this patient doesn’t meet our criteria for the weight management service’.” (F1)

“I still I think a lot of it is down to the communication aspect again and so I think that doing more face to face communication with people and raising awareness, so whether it's, you know, attending whatever kind of meetings so that you can have more of a conversation about it would be helpful from that point of view because I think, I do think, you know, email, etc. has its place and it is very useful but I don’t think anything, you know, kind of compares to face to face” (F4)

The second tension related to the *message* being communicated to primary care practitioners by the service, between stressing the importance of weight loss versus more holistic wellbeing messages. This, in turn, is likely to affect both how practitioners ‘sell’ the service to patients and patients’ expectations of the service. This was a tension felt most acutely by the service in health board G, which had adopted a *Health at every size* approach.

“We are now in the position to go and have a few more discussions with GPs because really what we don’t want is - because of the approach we take - we don’t want GPs to tell people to lose weight all the time.” (F7)

A key aspect of this tension is about shaping GP expectations of the service, by providing them with information about what is considered a good result. For the majority of services where weight loss was the ultimate goal, it was important to make referring practitioners aware of the realistic weight loss outcomes from the service.

“…in all our discharges we put on, ‘five kilogram weight [loss]’, and we reference SIGN [national guidelines], and ‘this is considered successful and a clinical improvement.’ And, we put it in every bit of our literature that we can, because that is an education to our referrers.” (F5)

Finally, there was an evident tension around the GP *responses* to attempts by weight management services at engagement with primary care. When asked about previous contact with primary care, the following exchange between two interviewees in health board A gives a sense of the challenge:

“I think it’s so variable. You know I think some of our lead GPs have been fantastic at opening the gates for us.” (F1)

“But then you get other GPs who say ‘well I’m not doing weight management until you give me money’, so it’s ‘give me money’.” (M1)

The second quote above refers to the Quality and Outcome Framework (QOF), which was a pay-for-performance system that was used in general practice in Scotland at the time of the interviews, but has since been replaced.

Responses to more proactive methods of GP engagement by different weight management services have also been mixed. One respondent described the challenge of getting a GP representative on a weight management group. Others described poor turnout by GPs at awareness-raising or training events that had been organised. The main explanation offered by interviewees for the resistance to primary care engagement with weight management is that GPs do not see it as part of their role.

“Many many people in primary care… didn’t see weight management as their business.” (F5)

## Discussion

### Main findings

This study highlights a number of challenges that health authorities face when planning and managing adult weight management services. There are challenges for the weight management services themselves, such as insecurity of funding, due in part to a lack of a powerful lobbying voice for more resources. These funding issues can, in turn, result in changes to available services, making it difficult for primary care practitioners to keep abreast of what is available and fostering a degree of apathy towards these services – what has been described as ‘change fatigue’ [14].

Other challenges relate to tensions within general practice – notably around the extent to which obesity is considered a medical versus a social problem, but also related to role responsibilities of GPs versus practice nurses. These tensions are compounded by sub-optimal communication between adult weight management services and primary care. There were mixed messages at times (e.g. weight loss versus wellbeing) and inconsistent attempts at building relationships between the services. This may reflect the recognised challenges of dealing with a condition such as obesity, combining an individual, often medicalised approach within primary care consultations with the wider considerations of providing a more holistic, community-based service [23, 24].

### What is already known

Obesity is widely recognised as a major public health issue, but has not been accorded the same level of priority in terms of funding as other public health issues related to health behaviours, such as smoking cessation [25, 26]. Primary care is well placed to support adults with obesity [12], yet there are a number of barriers to engagement with weight management by primary care practitioners, including lack of time in the consultation [7], lack of knowledge and confidence in discussing weight [8], perceptions of poor outcomes of interventions [8], fear of causing offence [9], and a belief that obesity is not a medical problem [10]. Most of the research on barriers to engagement with weight management has only involved GPs and practice nurses [7–10, 27]. Few studies have explored the views of those involved in planning and providing weight management services about the interface with primary care.

Researchers from the Counterweight Programme conducted a focus group study with 7 weight management advisers, presented alongside qualitative interviews with patients and practitioners [28]. In keeping with our findings, they reported that engagement with primary care staff was influenced not just by practitioners’ beliefs and attitudes and practice-level factors, but also by the way in which the service was initiated and implemented.

### What this study adds

This is the first qualitative interview study that we are aware of to explore the views of key stakeholders involved in the planning and delivery of adult weight management services about the role of primary care in adult weight management. The findings help us understand the marked variation in engagement with adult weight management in primary care. In particular, communication with primary care was seen as very important, with those services that had a previous history of working closely with practices benefitting from this improved relationship. This is a key message; it is therefore imperative that weight management services are supported in the more time consuming, but ultimately effective, role of developing local relationships with potential referrers to their service. This is especially important if the over-riding ethos of the service is one of wellbeing rather than weight loss. However, even when weight loss is important, time and effort is required to engage with practitioners and highlight what are realistic expectations of the service.

Finally, weight management services themselves need to secure mainstream funding in order to develop long-term, sustainable strategies of engagement and service delivery. Our results suggest that too much time is spent fire-fighting the implications of short-term funding rather than building relationships with practitioners who can help engage with and refer those who would most benefit from the services on offer.

### Limitations of this study

The main limitations of this qualitative study are that it is a small sample, which was recruited pragmatically, so findings may be biased by self-selection. Our recruitment strategy was to ask for service leads involved in the strategic delivery of adult weight management services to volunteer to be interviewed; 7 of the 8 largest health boards in Scotland took part and, in all cases, the service lead was a dietician. While it is possible that other health professionals may be involved at a similar level of service delivery and, arguably, would have brought a different perspective to our study, it does seem to indicate a clear role for dieticians in the strategic delivery of such services. Furthermore, participant validation was not obtained following analysis due to limited time and resources; this would have strengthened the reliability and validity of the findings [29]. Finally, it is important to note that GPs are not involved in commissioning adult weight management services in Scotland (there is no so-called ‘purchaser-provider split’ [30]), so relationships between frontline clinicians and weight management service providers may be different in other parts of the UK and elsewhere.

## Conclusions

Responses to the public health problem of obesity need to be multi-sectoral, but if primary care is to fulfil its potential in this area –to increase the identification and referral of appropriate patients to weight management services [31] – there needs to be better engagement by weight management services with primary care. Furthermore, the services need more secure, sustained funding. This will require more effective lobbying for resources, though it is not clear where this pressure will come from. One vision for a way forward has been to call for weight management to follow the example of smoking cessation in the UK, where there is a network of well-resourced NHS Stop Smoking Services, accessible via different means and in different locations [13]. The present study has, however, highlighted a number of tensions to be negotiated for this vision to become a reality.

## Additional file

Additional file 1: Theory-Driven Stakeholder Interviews Topic Guide. (DOCX 20 kb)

## Abbreviations

BMI: Body mass index
GP: General practitioner
HAES: Health at every size
NHS: National Health Service
OSOP: ‘One sheet of paper’
UK: United Kingdom
